# A Prebiotic Hydrogel to Prevent Cervical Cancer by Optimizing the Vaginal Ecology and Reducing Oncogenic Biological Risk Factors in Women Across Global Health Care Access

**DOI:** 10.1200/GO.22.44000

**Published:** 2022-05-05

**Authors:** Joanna Ellington, G. Dennis Clifton, Rayne D. Ellington-Lawrence, Vega Zamora De la Fuente, Raina Fichorova

**Affiliations:** ^1^ Glyciome, LLC, Post Falls, ID; ^2^ Genital Tract Biology, Department of ObGyn & Reproductive Biology, Brigham & Women's, Harvard Medical School, Boston, MA

## Abstract

Persistent oncogenic, high-risk human papillomavirus (hrHPV) is a leading cause of cervical cancer and driven by vaginal dysbiosis and mucosal irritation. There is a high need for a product to optimize vaginal ecology and decrease biological risk factors of hrHPV persistence and cervical cancer.

**METHODS:**

A prebiotic hydrogel (PreBioGyn) was designed for cervical cancer prevention to support the cervicovaginal ecology and anti-viral function. PreBioGyn was compared to over-the-counter (OTC) acidifying vaginal gels RepHresh and Trimosan for impact on: (1) in-vitro growth of L. crispatus—a Lactobacillus essential for the healthy vaginal biome; (2) inhibition of P. bivia—a pathobiont characteristic of vaginal dysbiosis and directly linked to hrHPV reactivation and cervical cancer spread; (3) mucosal irritation potential measured as daily mucus production by slugs (Arion lusitanicus) after 5-day intermittent product exposure; and (4) osmolality and mucoadhesion essential for compatibility with the cervicovaginal mucosal barrier (ability of gel to interdigitate with mucins in vivo).

**RESULTS:**

Exposure to PreBioGyn for 24 hours completely suppressed P. bivia while sustaining L. crispatus growth which was recovered at 100 ± 1%. Trimosan was bactericidal to L. crispatus within 4 hours incubation and RepHresh within 24 hours of incubation. The mucosal irritation potential of PreBioGyn equaled negative controls, whereas the OTC acidifying-gels resulted in greater irritation potential with > 2-fold mucus production versus the control (*P* < .05). The PreBioGyn formula was iso-osmotic to the human vagina (280 mOsmo/kg), while RepHresh was hyper- (1,400 mOsmo/kg) and Trimosan - hypoosmotic (81 mOsmo/kg). The mucoadhesion index of PreBioGyn (10 ± 2.0) was higher (*P* < .05) than that of RepHresh (1 ± 0.2) and Trimosan (1 ± 0.3).

**CONCLUSION:**

Our results provide preclinical evidence for the biocompatibility and safety of the Pre-BioGyn gel. The prebiotic PreBioGyn vaginal gel improved microbiota parameters essential for cervical cancer prevention, compared to leading OTC acidifying-gels. PreBioGyn may offer a simple, affordable user-directed cervical cancer prevention intervention.

